# Effects of Tea Powder on the Cooking Properties, Antioxidative Potential and Volatile Profiles of Dried Noodles

**DOI:** 10.3390/foods11060858

**Published:** 2022-03-17

**Authors:** Kayama Kayama, Ran Wei, Yuanping Zhang, Fenghua Wu, Zhucheng Su, Junjie Dong, Xingquan Liu

**Affiliations:** 1 Department of Food Science, Zhejiang Agriculture and Forestry University, Hangzhou 311300, China; junior.kayama2@gmail.com (K.K.); zhangyuanp06@163.com (Y.Z.); wufh@zafu.edu.cn (F.W.); 2 Department of Tea Science, Zhejiang Agriculture and Forestry University, Hangzhou 311300, China; zhuchengsu@zafu.edu.cn; 3 Zhejiang Camel Transworld Organic Food Co., Ltd., Hangzhou 310041, China; djj@organic-tea.com

**Keywords:** dried tea noodle, tea powder, catechin, antioxidant, volatile compounds

## Abstract

Numerous studies indicate that tea has versatile health benefits, and attempts are being made to use it as a food additive. In this study, three types of tea powder (TP) [matcha tea powder (MTP), green tea powder (GTP), and black tea powder (BTP)] were used in noodle processing, and the cooking properties, antioxidant potential, and volatile profiles of dried tea noodles (DTN) were investigated. Between 0.5% and 2% TP addition decreased the cooking time, cooking loss, and water absorption of DTN, regardless of concentrations. TP decreased the brightness (L*) of the DTN while increasing the greenness (|−a*|) and yellowness (b*) values of matcha tea noodles (MTN) and green tea noodles (GTN), as well as the redness (a*) and yellowness (b*) values of black tea noodles (BTN). The results of the 1,1-Diphenyl-2-Picrylhydrazyl (DPPH) scavenging activity (10.84–95%), 2,2′-azinobis-(3-ethylbenzothiazoline-6-sulfonic acid) (ABTS) free scavenging activity (2.03–92.23%), and total phenolic content (TPC) (97.32–540.97 mg/g) of the noodles increased as the TP addition increased. Besides, TP also enriched the flavor of the DTN, with alcohol, aldehydes, and ethers being the main components. In conclusion, the addition of TP positively improved the quality of the DTN and increased its antioxidative potential.

## 1. Introduction

Tea is an aromatic beverage commonly prepared by pouring hot or boiling water over cured or fresh leaves of *Camellia sinensis*, an evergreen shrub native to East Asia [1]. After water, it is the most widely consumed drink in the world [2]. Tea types, based on processing or harvested leaf development, are black (fermented), green (non-fermented), oolong (semi-fermented), etc., [3].

Tea has a historical reputation of being a healthy plant because of its various bioactive components, such as polyphenol (18–36% dry weight), amino acids (1–4% dry weight), caffeine (2–5%), etc., [4,5,6]. Several lines of scientific evidence suggest that tea possesses a wide range of biological activities, such as anti-obesity, anti-stroke, anti-cancer, anti-inflammation, etc., [7].

In the food industry recently, tea is usually pulverized into several micron granularity powders by ultra-micro pulverization technology or grinding, which can be directly used for eating or processing various snacks, beverages, bakery products, and so on. In this project, matcha, green tea powder, and black tea powder were selected as the nutritional subjects to be used to produce noodles. Since tea is still one of the most consumed beverages in the world, the health effects of consuming this beverage seem to be particularly relevant to testing.

Wheat flour noodles are a crucial part of the diet of many Asians. It is believed that noodles originated in China as early as 5000 BC then spread to other countries [8]. Through fortification, many researchers have now concentrated on the quality improvement of noodles by obtaining stable noodles with high nutritional values and health benefits. Fortification of foods has been utilized by the food industry for years and continues to be beneficial in providing consumers with added nutrition. The World Health Organization (WHO) and the United States Food and Drug Administration (FDA) consider noodles to be a good product for nutritional improvement due to their low levels of protein and fiber [9].

Xu et al. [10] studied the evaluation of different tea extracts on the dough, textural, and functional properties of dry Chinese white salted noodles with enhanced antioxidant capacity. The results showed that the amount of tea extract and the type affected the mixing characteristics of the dough, as well as the subsequent noodle color, cooking yield, and the texture of the cooked noodles. At the same time, as the addition of tea extract increased from 0.0 to 2.0%, the total phenolic content of noodles, 1,1-diphenyl-2-picryl hydrazyl (DPPH) free radical clearance activity, and resistant starch content increased. Research on the water cooking stability of dried noodles enriched with different particle sizes and concentrations of green tea powders by Yu et al. [11] found that the antioxidant activity and phenolic compounds of dry green tea noodles decreased significantly after water cooking. The results showed that large green tea granules increased the cooking loss of green tea noodles, but the phenolic compounds of dry green tea noodles prepared with them were lost after cooking. Texture properties and microstructure analysis showed that large green tea powder particles with a concentration of 2% formed some holes in the noodle network, and their breaking strength decreased.

However, the effect of different types of tea powder on dried noodles remains unclear. The aim of this study is to explore the cooking properties, color, antioxidant activity, and volatile profile of DTN incorporated with matcha, green, or black tea powder and provide a theoretical basis for the food industry.

## 2. Materials and Methods

### 2.1. Materials

Matcha, green tea powder, and black tea powder were purchased from Camel Transworld Co., Ltd. (Hangzhou, China). Wheat flour was purchased at a local supermarket (Hangzhou, China). Additionally, 1,1-diphenyl-2-picryl hydrazyl (DPPH), 2,2′-Azinobis-(3-ethylbenzthiazoline-6-sulphonate) (ABTS), and catechins standards for high-performance liquid chromatography (HPLC) were purchased from Yuanye Bio-Technology Co., Ltd. (Shanghai, China). Other reagents used in the analytical procedures were purchased from Sinopharm Chemical Reagent Co., Ltd. (Shanghai, China).

### 2.2. Chemical Composition of Tea Powder and Wheat Flour

Extraction of TP (MTP, GTP, and BTP) was performed according to Ying et al. [12] with some modifications. Briefly, 1.5 g of tea powder was ultrasonically extracted twice with 250 mL of boiling distilled water in a boiling water bath for 1 h. Infusions were mixed and quickly filtered. Total phenolic content (TPC) in tea samples (MTP, GTP, and BTP) was measured following the Folin–Ciocalteu procedure of Xu et al. [10], with gallic acid as the standard. Briefly, 1 mL of the sample extract was mixed with 5 mL of Folin–Ciocalteu reagent. For 10 min, the mixture was constantly stirred, and then 4 mL of sodium carbonate solution and 2 mL of distilled water were added. The absorbance of the mixture was measured with a spectrophotometer (INESA UV752N, Shanghai, China) at 750 nm. Amino acid content was measured using nihydrin colorimetry method [13] with a standard of theanine. In brief, TP extracts (1 mL), phosphate buffer (0.5 mL), and a 2% nihydrin solution (0.5 mL) were mixed together. The total mixture was heated in a boiling water bath for 15 min. After it had cooled to room temperature, distilled water was added to get it to 25 mL, and then absorbance at 570 nm was measured. The total soluble sugar content was determined by the method of Morris et al. [14], with glucose as standard. Briefly, 1 mL of the sample extract was pipetted into a 10-mL test tube, along with 5 mL of anthrone reagent, and was well mixed by vortexing. The mixture was heated in a water bath for 8 min and then cooled at room temperature. Absorbance at 630 nm was measured. The analysis of catechins and caffeine content in the tea samples was performed by HPLC on a SHIMADZU LC-2010A HPLC system (Shimadzu Corporation, Tokyo, Japan). Chromatographic conditions: C18 inertsil ods-sp column (250 mm × 4.6 mm, 5 μm); Mobile phase A: 0.1 mol/L sodium acetate buffer acetonitrile (97: 3, *v/v*); Mobile phase B: acetonitrile water (4: 1, *v/v*); Flow rate: 1.0 mL/min; Detection wavelength: 254 nm; Column temperature: 35 °C; Injection volume: 10 μL. All chemical standards were of high-performance liquid chromatography grade.

Proximate compositions (protein, fat, gluten, ash, and moisture) of wheat flour were tested by using the standard methods of the Association of Official Analytical Chemists (AOAC) [15].

### 2.3. Dried Tea Noodle Production

DTN were prepared following the method described by Parvin et al. [16]. The basic noodle formula consisted of 100 g of wheat flour, 45 mL of distilled water, and 1 g of salt. Experimental noodles were prepared by substituting wheat flour with 0.5%, 1%, 1.5%, and 2% of MTP, GTP and BTP, and were marked as CTRL (control noodle sample without tea powder), M 0.5%, M 1.0%, M 1.5%, M 2.0%, G 0.5%, G 1.0%, G 1.5%, G 2.0%, B 0.5%, B 1.0%, B 1.5%, and B 2.0% (M: MTN, G: GTN, and B: BTN), respectively. The dough was formed using a vacuum mixer, all ingredients were added into a vacuum mixer to mix, while water was added slowly while mixing. The mixing time was 15 min for each sample. After mixing, the dough was wrapped in plastic and rested for 30 min. The dough was compressed to form a continuous dough sheet of 1.5 mm thickness, which was passed through subsequent rolls. After sheeting, the dough sheet was cut into noodle strands and was dried for 3 h at 55 °C using the oven (heat) drying method. After drying, the noodles were packed and stored in the refrigerator at 4 °C for further analysis.

### 2.4. Cooking Properties

The cooking time and cooking loss of DTN samples were measured according to the guidelines of the American Association of Cereal Chemists (AACC 66–50) [17]. Briefly, 20 g DTN was placed into 400 mL of boiling water. A noodle strand was removed every 30 s and squeezed between 2 pieces of clear plastic. The optimal cooking time was determined when the white center core just disappeared. To determine cooking loss, 5 g of DTN was placed into 500 mL of boiling distilled water. Then, we quantitatively transferred the cooking/rinse water to the beaker and evaporated to dryness in an oven at 105 °C. After drying, the beakers were allowed to cool to room temperature before being weighed. Water absorption of DTNs was determined according to the method by Sirichokworrakit et al. [18]; 20 g of DTN sample was placed into 400 mL of boiling water at the previously determined cooking time. After boiling, the noodles were removed and weighed. The weight difference between dried noodles and cooked noodles was used to calculate the water absorption.

### 2.5. Color Analysis

To determine the color of the noodles, color values were obtained by using the color reader (Model CR-10, Konica Minolta, Inc., Tokyo, Japan). Readings (L*, a*, and b*) were taken from each noodle sample after placing the color reader on the noodle sample. L* is the brightness; for a*, negative values indicate greenness and positive values indicate redness; for b*, negative values indicate blueness and positive values indicate yellowness.

### 2.6. Antioxidant Activity

The DPPH and ABTS scavenging activity methods were used to evaluate the antioxidant activity of the DTN. DPPH assay was measured according to the method of Lee et al. [19]. Briefly, 0.2 mL of DTN extracts were combined with 1 mL of 0.041 mM DPPH in ethanol. The mixture was vigorously mixed and allowed to stand at room temperature for 30 min before measuring absorbance at 517 nm. ABTS assay was measured according to the method of Re et al. [20]. In brief, to prepare the ABTS^+^ solution, 38.4 mg of ABTS was dissolved in 10 mL 2.5 mM potassium persulfate, and the mixture was stored in the dark at room temperature for 12–16 h. ABTS^+^ solution was diluted with ethanol and distilled water to achieve an absorbance of 0.70 ± 0.02 at 734 nm. A total of 100 μL of the sample extract was mixed thoroughly with 3.9 mL of prediluted ABTS^+^ (absorbance 0.70 ± 0.02) by vortexing. The reaction mixture was incubated at room temperature for 2 to 10 min before the absorbance at 734 nm was measured.

### 2.7. Determination of Volatile Compounds

Volatile compounds of DTN were determined according to Antoniewska et al. [21]. Headspace solid-phase micro-extraction (SPME) coupled with gas chromatography-mass spectrometry (GC/MS) SHIMADZU GC-MS-QP2010 (Shimadzu Enterprise Management, Nagano, Japan) were used to determine the volatile compounds in DTN. Cooked DTN (0.5 g) were placed in a headspace vial glass bottle. The headspace bottle was placed in a constant temperature water bath at 60 °C for 20 min, then the extraction head was inserted for 40 min, and the sample was desorbed at the injection port at 250 °C for 5 min, then analyzed by GC-MS.

GC conditions: DB-Wax capillary column (30 cm × 0.25 mm, 0.25 μm); carrier gas is helium, flow rate 1.2 mL/min, splitless injection; temperature rise program: initial temperature 40 °C, hold for 3 min, raise the temperature to 200 °C at 5 °C/min, then raise it to 230 °C at 10 °C/min, and keep it for 3 min. MS conditions: Electron ionization source; electron energy 70 eV; transmission line temperature 280 °C; ion source temperature 230 °C; quadrupole temperature 150 °C; mass scanning range *m*/*z* 55~500.

### 2.8. Statistical Analysis

All experimental measurements were carried out in triplicate, and the results are expressed as mean ± standard deviation (SD). The significant differences were checked by a one-way ANOVA test using SPSS 28.0 (IBM, Chicago, IL, USA); the value of *p* < 0.05 was deemed statistically significant. All figures were drawn by Microsoft Excel 2010 (Microsoft, Redmond, WA, USA).

## 3. Results

### 3.1. Chemical Composition of Tea Powder and Wheat Flour

The major components of tea powder samples (MTP, GTP, and BTP) were evaluated and are shown in Table 1 and Table 2. The proximate composition of wheat flour is presented in Table 3. Wheat flour provided 13.91%, 0.5%, 11.99%, 30.24%, and 1.38% of moisture, ash, protein, gluten, and fat contents, respectively. As presented in Table 1, there were significant differences in the moisture, caffeine, and soluble sugars content of all the TP samples. On the other hand, amino acids appeared at a major percentage in MTP (9.3%), followed by GTP (2.4%), and BTP (1.5%). GTP (14.1%) recorded a higher content in tea polyphenol than MTP (10.45%) and BTP (7.4%). HPLC analysis was used to determine the amount of catechin monomers and caffeine in the tea powder samples. The major catechin corresponded to EGCG, whose amount reached up to 75.84 mg/g in the GTP sample. GC is the catechin that appeared with the major amount in the BTP (51.85 mg/g) sample; this amount was higher than the corresponding values in MTP (13.12 mg/g) and GTP (44.80 mg/g). EC, GCG, ECG, and CG recorded higher amounts in GTP than the corresponding values in MTP and BTP. EGC recorded high amounts in MTP, followed by GTP, and then BTP.

### 3.2. Cooking Properties

The results of the cooking properties of DTN, which are the cooking time, water absorption, and cooking loss, are shown in Table 4. Compared to the CTRL, adding MTP, GTP, and BTP significantly decreased the optimal cooking time. The cooking time for the CTRL noodle was 12.75 min, and it decreased to 11.5–10.25 min for 0.5–2% MTN addition, 11.5–9.25 min for 0.5–2% GTN addition, and 11.25–9.75 min for 0.5–2% BTN addition. The results also show that there was a decrease in water absorption. The water absorbance was 146.5% for the CTRL noodle, and it decreased to 83.5–138.7% for 0.5–2% MTN addition, 111–141.5% for 0.5–2% GTN addition, and 76.5–135.5% for 0.5–2% BTN addition. It can be seen that cooking loss was a maximum of 7% in the CTRL sample. After 0.5–2% TP (MTP, GTP, and BTP) addition the cooking loss decreased to 5.0–6.0%. There was a significant reduction in cooking loss with the addition of TP, while the concentration of the TP did not affect the cooking loss.

### 3.3. Color Analysis

**Figure 1:**
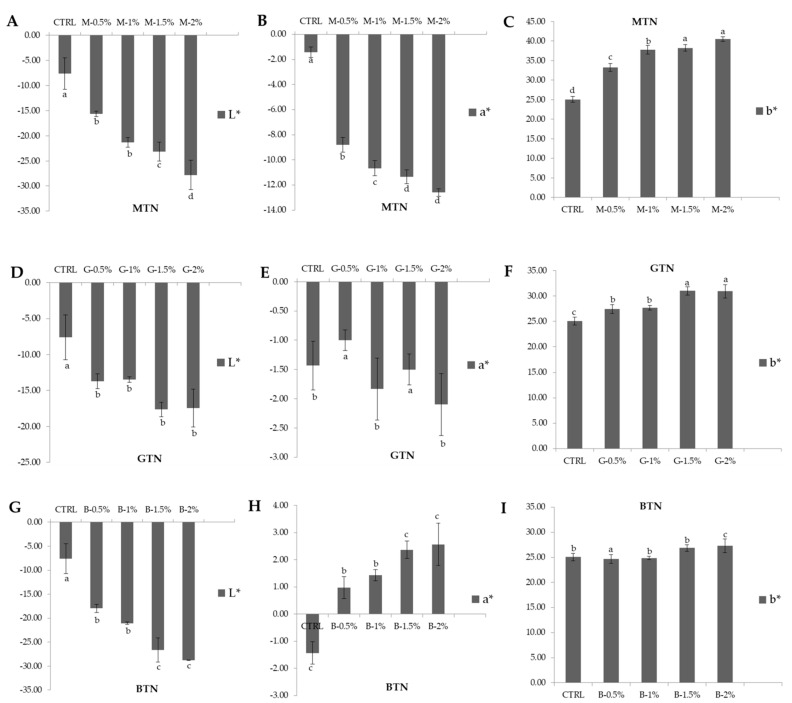
Color analysis of noodle samples: (**A**) Matcha tea noodles (MTN) brightness (L*) in color; (**B**) Matcha tea noodles (MTN) greenness (-a*) in color; (**C**) Matcha tea noodles (MTN) yellowness (b*) in color; (**D**) Green tea noodles (GTN) brightness (L*) in color; (**E**) Green tea noodles (GTN) greenness (-a*) in color; (**F**) Green tea noodles (GTN) yellowness (b*) in color; (**G**) Black tea noodles (BTN) brightness (L*) in color; (**H**) Black tea noodles (BTN) redness (a*) in color; (**I**) Black tea noodles (BTN) yellowness (b*) in color. CTRL: noodles without tea powder; M-0.5%, M-1.0%, M-1.5%, and M-2.0%: noodles with additional 0.5%, 1.0%, 1.5%, and 2.0% matcha tea powder, respectively; G-0.5%, G-1.0%, G-1.5%, and G-2.0%: noodles with additional 0.5%, 1.0%, 1.5%, and 2.0% green tea powder, respectively; B-0.5%, B-1.0%, B-1.5%, and B-2.0%: noodles with additional 0.5%, 1.0%, 1.5%, and 2.0% black tea powder, respectively; All addition levels were on a flour basis. The values followed by different letters were significantly different (*p* < 0.05).

Comparisons of noodle color between the CTRL (noodles without tea powder) with MTN, GTN, and BTN are shown in Figure 1. The results showed that the L* (brightness) of MTN and GTN decreased, and |-a*| (greenness) values increased as the amount of TP increased in noodles, while the b* (yellowness) values increased. In BTN, L* values decreased, while a* (redness) and b* (yellowness) values increased, suggesting that the addition of TP reduced the brightness of the noodles compared to the CTRL noodle sample.

### 3.4. Antioxidant Activity

**Figure 2:**
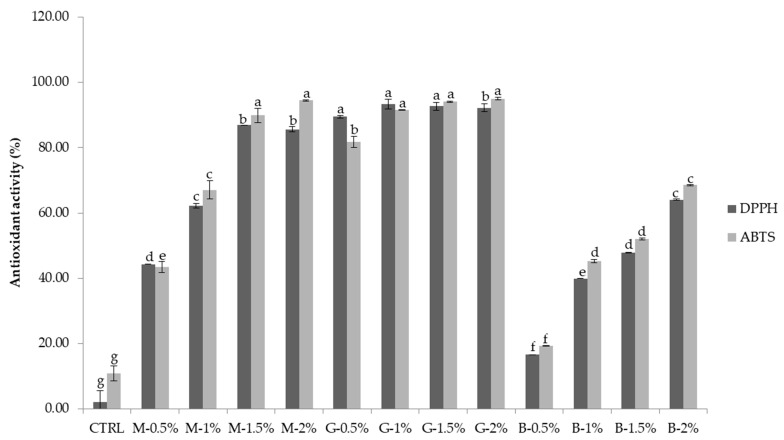
Antioxidant activity (% of control) of dry tea noodles with different tea extracts. CTRL: noodles without tea powder; M-0.5%, M-1.0%, M-1.5%, and M-2.0%: noodles with additional 0.5%, 1.0%, 1.5%, and 2.0% matcha tea powder, respectively; G-0.5%, G-1.0%, G-1.5%, and G-2.0%: noodles with additional 0.5%, 1.0%, 1.5%, and 2.0% green tea powder, respectively; B-0.5%, B-1.0%, B-1.5%, and B-2.0%: noodles with additional 0.5%, 1.0%, 1.5%, and 2.0% black tea powder, respectively; All addition levels were on a flour basis. The values followed by different letters were significantly different (*p* < 0.05).

**Figure 3:**
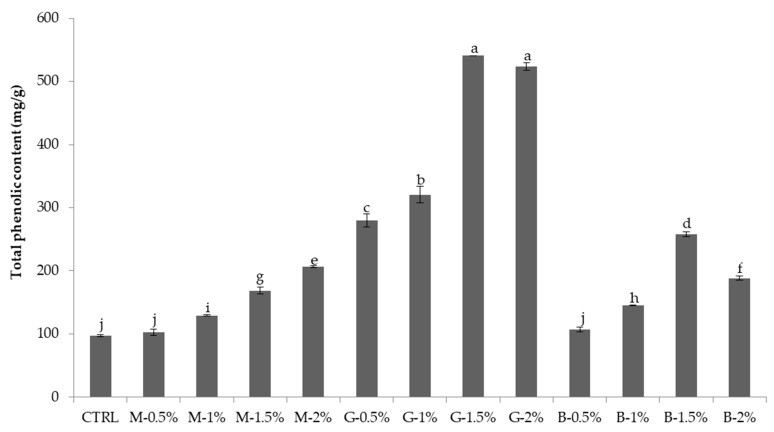
Total phenol content (TPC) of dry tea noodles with different tea extracts. CTRL: noodles without tea powder; M-0.5%, M-1.0%, M-1.5%, and M-2.0%: noodles with additional 0.5%, 1.0%, 1.5%, and 2.0% matcha tea powder, respectively; G-0.5%, G-1.0%, G-1.5%, and G-2.0%: noodles with additional 0.5%, 1.0%, 1.5%, and 2.0% green tea powder, respectively; B-0.5%, B-1.0%, B-1.5%, and B-2.0%: noodles with additional 0.5%, 1.0%, 1.5%, and 2.0% black tea powder, respectively; All addition levels were on a flour basis. The values followed by different letters were significantly different (*p* < 0.05).

Comparisons in the antioxidant activity of MTN, GTN, and BTN determined by two different methods (DPPH and ABTS scavenging activity) are shown in Figure 2. The enrichment of noodles with MTP, GTP, and BTP (0–2%) resulted in a significant increase in antioxidant properties compared with the control sample. The DPPH values increased from 2.03% to 85.64% (0.5–2%: MTN addition), 92.23% (0.5–2%: GTN addition), and 64.08% (0.5–2%: BTN addition), respectively. The ABTS values increased from 10.84% to 94.4% (0.5–2%: MTN addition), 95.0% (0.5–2%: GTN addition), and 68.48% (0.5–2%: BTN addition), respectively. The TPC in DTN samples ranged from 97.32 mg/g to 540.97 mg/g (Figure 3). GTN recoded the highest TPC compared to MTP and BTP, with the highest values at G 1.5% (540.97 mg/g) and G 2% (523.42%), as shown in Figure 3.

### 3.5. Determination of Volatile Compounds

The analysis of volatile compounds in the headspace of MTN, GTN, and BTN using SPME GC-MS yielded a total of 32 identified and quantified compounds (Table 5). The identified volatile compounds consisted of 9 alcohols, 8 aldehydes, 6 ethers, 1 fatty acid, 1 hydrocarbon, 1 ketone, 1 heteroaromatic, and others. Additionally, 1-heptanol, Hexanol, 1-pentanol, Benzaldehyde, Hexanal, 1,4,7,10,13,16-hexaoxacyclooctadecane, Methoxybenzoxime, and 6-Methyl-5-hepten-2-one were present in all the samples.

## 4. Discussion

In the present study, TP supplementation significantly reduced cooking loss, cooking time, and water absorption, regardless of the type of TP used. It also enriched the color and flavor and increased the antioxidant activity and TPC of DTN, which makes tea a potential food additive.

After analyzing the chemical composition of MTP, GTP, and BTP, the results showed that the content of tea polyphenols in MTP was lower than that in GTP; similar results were reported by Nishitani et al. [22]. It was then suggested that such a result might be due to the shade cultivation of its leaves, which reduces the biosynthesis of catechins. BTP had the lowest concentration of tea polyphenols; this is because, in black tea, some tea polyphenols are significantly reduced during the fermentation process and are, therefore, either absent or present in low levels [23]. Caffeine concentration in GTP (2.32%) was comparable to MTP (2.18%) and BTP (2.10%). MTP (9.3%) had the highest percentage of amino acids, while GTP (2.4%) and BTP had the lowest (1.5%). This is due to the fact that the green tea leaves used to make matcha are grown in the shade, which prevents amino acids from breaking down. As a result, Tencha leaves contain more of it than other teas [24]. The relatively high content of amino acids in matcha is the reason for its unique non-bitter taste [25], thus it would be good for noodle production. Noodles are made from semolina and various flours, but soft white wheat flour is usually the first choice. If high strong protein flour is used, the noodles will have too much elasticity and chewiness during cooking [26], thus soft white wheat flour was used in this study.

The cooking properties of DTN, which are the cooking time, water absorption, and cooking loss, were analyzed. As the amount of tea powder increased, the optimal cooking time decreased. This could be due to gluten dilution in the dough. Gluten is primarily responsible for the formation of a starch/protein complex, which determines the structure and cooking characteristics of noodles. By diluting these ingredients with tea powder, you may be able to shorten the cooking time. [27]. The decrease in cooking time of the DTN is in accordance with a study by Chillo et al. [28], who attributed the increase in the speed of water penetration of the gluten matrix to the addition of non-gluten or less gluten. The results are inconsistent with those described by Petitot et al. [29] and Kushtova et al. [30]. Padalino et al. [31] observed the reduction in cooking time in the fortification of tomato peel flour in pasta. Results indicated that there was a decrease in water absorption, and this phenomenon may be due to the presence of the lower amount of hydrophilic constituents in the tea powders or the water competition effect between hydroxyl groups of TPs and starch. These results are also in agreement with [10,11,32]. There was a significant reduction in cooking loss with the addition of tea powder, while the concentration of the tea powder did not affect cooking loss. This may be due to the better combination of starch granules and tea powder added to the gluten matrix [27]. The results are in alignment with those described by Rekha et al. [27]. However, the results are not in agreement with those found by Yu et al. [11]. The results showed that the cooking loss of dried green tea powder noodles significantly increased with the increase in GTP particle size; this might be due to the large particle size of GTP, which could weaken the network of noodles, increasing starch granule loss, and it might also be due to the pregelatinized effect of tea powders by extrusion cooking treatment, which can modify the starch structure of tea powder and pasting properties of wheat flour [11].

Noodle color is one of the most important factors in determining consumer acceptance. According to Takata et al. [33], the flour content is often influenced by its polyphenolic content, and, since tea is characterized mainly by polyphenols, the change in the color of the noodles is attributed to the addition of tea powders to the wheat flour. Xu et al. [10] reported that green tea extract was yellowish-green with L*, a*, and b*, completely different from flour. The orange-red of black tea extract and oolong tea extract had different L* and a* but had similar b* values as flour. The color difference between tea extract and control flour changed the color of fresh noodles.

The enrichment of noodles with MTP, GTP, and BTP resulted in an increase in antioxidant properties compared with the CTRL. Figure 1 shows that the percent inhibition of DPPH and ABTS increases with the increase in the amount of tea powder in wheat flour. The enhanced antioxidant properties of tea noodles were due to the presence of phenolic compounds in the tea extract. The DPPH results were consistent with the reported study of Ahmad et al. [32]. The TPC in the examined extracts ranged from 97.32 mg/g to 540.97 mg/g. GTN recoded the highest TPC compared to MTP and BTP. The results are similar to those found by Xu et al. [10], where, at 0.5% and 1% addition levels, noodles incorporated with green tea showed a higher TPC than with BTN and OTN. The result may be due to the interaction between the catechins mainly contained in GTP and the macromolecules in flour. The DTN with higher flavonoid content and antioxidant abilities might have health benefits for people since some researchers reported that flavonoids had antioxidative, anticarcinogenic, anti-inflammatory, antiaggregatory, and vasodilatory effects, and they have been linked to the prevention of various chronic diseases [34].

The data obtained indicated that alcohols, aldehydes, and esters were the dominant volatile compounds in DTN. GTN had the highest percentage of alcohol, followed by MTN and BTN. Among the alcohol compounds, 1-octen-3-ol was identified as one of the major volatile compounds of DTN, with GTN having the highest concentration (at G2%: 17.7%). Alcohols were the primary source of aroma in DTN due to the sensitive perception of odor; they produce a pleasant aroma that can be described as sweet, floral, or fruity [35]. So far, there is no study on the determination of volatile compounds in DTN; most researchers have rather focused on the determination of volatile compounds in wheat flour, wheat flour noodles, or tea. However, this study has many volatile compounds in common with some researchers [36,37,38,39] who have focused on the determination of volatile compounds in teas, wheat flour, and wheat flour noodles.

A total of 8 different aldehydes were identified, although not all aldehydes were found in all samples. The aldehyde hexanal, which was the major volatile compound in DTNs, is characterized by a low threshold odor that is described as grassy and hay-like [40,41]. Hexanal was present in all DTN samples; however, the CTRL recorded the highest concentration. Lee et al. [37] studied the volatile profile of 24 green tea samples and reported that hexanal was found in almost all the green tea samples. Lee et al. [37] also described the aroma of hexanal as being similar to freshly cut grass and unripe fruits in extreme dilution. Yang et al. [38] studied the volatile profile of green tea; they also reported most of the aldehydes as in our study, where volatile compounds, such as heptanal and benzaldehyde, were reported. Aldehydes play an important role in the entire odor because of their relatively low odor threshold values [42]. According to Yang et al. [38], benzaldehyde, which was present in all our DTN samples, is typically described as fragrant, sweet, and having an almond aroma; thus, it can be good for the noodles. Benzaldehyde was also identified in green tea by Lee et al. [37].

Ethers were the third major chemical group reported in our study. Overall, 1,4,7,10,13,16-hexaoxacyclooctadecane was the major ether compound in DTNs, with the highest concentration in BTN (at B2%; 7.48%), followed by methoxybenzoxime, and others. Ketones have some impact on the volatility profile of DTNs, too. Only one ketone (6-methyl-5-hepten-2-one) was detected in this study, and it was present in all samples. Wu et al. [39] studied the volatile compounds of different fresh wet noodle cultivars and reported that 6-Methyl-5-hepten-2-one had a citrus odor and was detected in almost all the samples. Volatile hydrocarbons 3-ethyl-2-methyl-1,3-hexadiene was detected in substantial amounts. This might be due to tea polyphenols in the tea powder noodles [43].

## 5. Conclusions

Our research looked into the idea of making acceptable noodles out of wheat flour combined with various degrees of matcha, green, or black tea powder. The properties of cooking, antioxidant capacity, and volatile compounds were examined. The addition of tea powder to wheat flour changed the overall qualities of the noodles. The cooking properties of the noodles improved when they were reinforced, as shown by metrics, such as water absorption and cooking loss. Furthermore, compared to the control sample, antioxidant capacity findings revealed that all samples had significantly greater radical scavenging abilities. Thus, the above study could lead to new ways to prepare noodles with 0.5–2% of tea powder, which greatly impacts the basic quality and functional properties of noodles.

## Figures and Tables

**Table 1:** Chemical analysis of MTP, BTP, and GTP.

	MTP	GTP	BTP
Amino acids (%)	9.30 ± 0.22 ^a^	2.40 ± 0.35 ^b^	1.50 ± 0.06 ^b^
Tea polyphenols (%)	10.45 ± 1.35 ^b^	14.10 ± 0.58 ^a^	7.40 ± 0.32 ^c^
Soluble sugar (%)	1.75 ± 0.05 ^a^	1.98 ± 0.27 ^a^	1.99 ± 0.04 ^a^
Caffeine (%)	2.18 ± 0.13 ^a^	2.32 ± 0.54 ^a^	2.10 ± 0.07 ^a^
Moisture (%)	5.00 ± 0.02 ^a^	4.00 ± 0.19 ^b^	6.00 ± 1.03 ^a^

MTP; matcha tea powder, GTP; green tea powder, BTP; black tea powder. Results are presented as means ± standard deviations (*n* = 3). Means with different small letter (a–c) superscripts in the same column are significantly different at *p* < 0.05.

**Table 2:** Content of catechin monomers (mg/g) from MTP, BTP, and GTP.

Compounds (mg/g)	MTP	GTP	BTP
GC	13.12 ± 0.32 ^c^	44.80 ± 0.79 ^b^	51.85 ± 0.24 ^a^
EGC	14.92 ± 0.24 ^a^	7.68 ± 0.02 ^b^	2.74 ± 0.01 ^c^
C	0.70 ± 0.01 ^b^	61.87 ± 0.33 ^a^	0.89 ± 0.00 ^b^
EC	3.60 ± 0.21 ^b^	27.30 ± 0.04 ^a^	1.61 ± 0.23 ^b^
EGCG	66.18 ± 0.12 ^b^	75.84 ± 0.05 ^a^	3.65 ± 0.46 ^c^
GCG	2.91 ± 0.06 ^b^	6.97 ± 0.23 ^a^	0.54 ± 0.11 ^c^
ECG	1.10 ± 0.09 ^b^	2.17 ± 0.05 ^a^	0.56 ± 0.02 ^c^
CG	10.75 ± 0.12 ^b^	58.86 ± 0.23 ^a^	1.70 ± 0.00 ^c^

MTP; matcha tea powder, GTP; green tea powder, BTP; black tea powder. GC, (+)-gallocatechin; EGC, (−)-epigallocatechin; C, (+)-catechin; EC, (−)-epicatechin; EGCG, (−)-epigallocatechingallate; GCG, (+)-gallocatechingallate; ECG, (−)-epicatechingallate; CG, (−)-epigallocatechingallate. Results are presented as means ± standard deviations (*n* = 3). Means with different small letter (a–c) superscripts in the same column are significantly different at *p* < 0.05.

**Table 3:** Proximate composition of wheat flour.

	Moisture (%)	Ash (%)	Protein (%)	Gluten (%)	Fat (%)
Wheat flour	13.91 ± 0.01	0.50 ± 0.06	11.99 ± 0.06	30.24 ± 0.26	1.38 ± 0.07

Results are presented as means ± standard deviations (*n* = 3).

**Table 4:** Cooking properties of DTN.

Sample	CT (min)	CL (%)	WAC (%)	MC (%)
CTRL	12.75 ± 0.25 ^a^	7.00 ± 4.24 ^a^	146.50 ± 2.12 ^a^	10.14 ± 0.04 ^d^
M-0.5%	11.50 ± 0.50 ^a^	6.00 ± 5.65 ^a^	83.50 ± 16.26 ^e^	11.67 ± 0.05 ^c^
M-1.0%	11.25 ± 0.25 ^b^	6.00 ± 2.82 ^a^	105.25 ± 19.44 ^c^	11.77 ± 0.07 ^a^
M-1.5%	10.50 ± 0.00 ^b^	5.00 ± 4.24 ^a^	113.65 ± 16.05 ^a^	11.84 ± 0.02 ^b^
M-2.0%	10.25 ± 0.25 ^c^	6.00 ± 2.82 ^a^	138.70 ± 5.23 ^b^	11.85 ± 0.04 ^b^
G-0.5%	11.50 ± 0.00 ^b^	5.00 ± 1.41 ^a^	111.00 ± 1.41 ^d^	11.65 ± 0.04 ^c^
G-1.0%	11.00 ± 0.00 ^c^	6.00 ± 2.82 ^a^	120.00 ± 1.41 ^c^	11.75 ± 0.08 ^b^
G-1.5%	10.25 ± 0.25 ^d^	5.00 ± 1.41 ^a^	128.50 ± 2.12 ^a^	11.91 ± 0.01 ^a^
G-2.0%	9.25 ± 0.25 ^e^	6.00 ± 2.82 ^a^	141.50 ± 0.70 ^b^	11.96 ± 0.01 ^a^
B-0.5%	11.25 ± 0.25 ^c^	5.00 ± 4.24 ^a^	76.50 ± 2.12 ^f^	11.50 ± 0.07 ^c^
B-1.0%	10.75 ± 0.25 ^d^	5.00 ± 1.41 ^a^	102.00 ± 1.41 ^e^	11.64 ± 0.09 ^b^
B-1.5%	10.00 ± 0.00 ^e^	6.00 ± 2.82 ^a^	110.50 ± 2.12 ^b^	11.72 ± 0.09 ^c^
B-2.0%	9.75 ± 0.25 ^e^	5.00 ± 1.41 ^a^	135.00 ± 2.82 ^d^	11.95 ± 0.04 ^a^

CT—Cooking time, CL—Cooking loss, WAC—Water absorption capacity, MC—Moisture content, M—Matcha, G—Green Tea powder, B—Black Tea powder; enriched (0.5–2%). CTRL: noodles without tea powder; M-0.5%, M-1.0%, M-1.5%, and M-2.0%: noodles with additional 0.5%, 1.0%, 1.5%, and 2.0% matcha tea powder, respectively; G-0.5%, G-1.0%, G-1.5%, and G-2.0%: noodles with additional 0.5%, 1.0%, 1.5%, and 2.0% green tea powder, respectively; B-0.5%, B-1.0%, B-1.5%, and B-2.0%: noodles with additional 0.5%, 1.0%, 1.5%, and 2.0% black tea powder, respectively; All addition levels were on a flour basis. Results are presented as means ± standard deviations (*n* = 2). Means with different small letter (a–f) superscripts in the same column are significantly different at *p* < 0.05.

**Table 5:** Gas Chromatography-Mass Spectrometry (GC-MS) analysis results of volatile compounds in DTN samples.

Volatile Compound (%)	Cc	DTN (%)												
		CTRL	M-0.5%	M-1%	M-1.5%	M-2%	G-0.5%	G-1%	G-1.5%	G-2%	B-0.5%	B-1%	B-1.5%	B-2%
1-heptanol	A	0.95 ± 0.00	1.70 ± 0.52	1.41 ± 0.21	1.45 ± 0.21	1.69 ± 0.67	1.27 ± 0.16	1.35 ± 0.13	1.36 ± 0.13	1.17 ± 0.31	1.43 ± 0.63	1.01 ± 0.43	1.09 ± 0.14	1.93 ± 0.35
Hexanol	A	5.37 ± 0.03	6.67 ± 0.77	6.52 ± 0.79	7.48 ± 1.02	9.38 ± 2.35	8.05 ± 1.91	8.71 ± 0.81	7.65 ± 0.40	6.45 ± 0.04	9.40 ± 1.15	8.51 ± 0.58	7.70 ± 1.39	8.09 ± 0.16
Isooctyl alcohol	A	1.78 ± 0.56	1.66 ± 0.20	1.85 ± 0.07	1.66 ± 0.50	2.39 ± 0.09	n.d	1.76 ± 0.06	1.84 ± 0.03	1.66 ± 0.05	2.08 ± 0.03	1.36 ± 0.03	1.47 ± 0.09	2.10 ± 0.00
1-Octen-3-ol	A	3.02 ± 0.01	n.d	11.09 ± 0.05	12.31 ± 1.41	8.91 ± 0.24	7.73 ± 1.76	13.26 ± 2.23	14.77 ± 0.02	17.74 ± 1.45	4.86 ± 0.40	3.49 ± 0.33	3.41 ± 0.59	4.73 ± 0.94
1-pentanol	A	3.23 ± 1.50	3.86 ± 0.23	4.94 ± 0.64	4.27 ± 1.32	7.74 ± 1.35	3.97 ± 0.56	4.83 ± 1.05	4.24 ± 0.05	4.63 ± 0.02	4.00 ± 0.64	3.61 ± 0.63	2.95 ± 0.35	4.17 ± 1.09
(Z)-3-hexen-1-ol	A	n.d	1.84 ± 0.37	3.42 ± 0.21	4.46 ± 1.28	8.09 ± 1.78	1.46 ± 0.23	1.44 ± 0.04	1.27 ± 0.33	1.64 ± 0.12	1.50 ± 0.02	1.79 ± 0.01	2.29 ± 0.25	3.11 ± 0.39
3-Octen-2-ol, (Z)-	A	1.09 ± 0.09	n.d	1.57 ± 0.29	n.d	1.98 ± 0.27	1.38 ± 0.56	1.48 ± 0.21	1.41 ± 0.02	1.21 ± 0.45	1.39 ± 0.67	1.13 ± 0.69	n.d	2.24 ± 0.70
(18S,19S)-18,19-Dihydroxy-1,4,7,10,13,16-hexaoxocycloencosane	A	1.21 ± 0.78	1.69 ± 0.28	3.60 ± 0.28	n.d	3.21 ± 0.72	n.d	n.d	1.44 ± 0.27	1.87 ± 0.25	n.d	n.d	3.19 ± 0.16	n.d
Linalool	A	n.d	n.d	n.d	n.d	n.d	1.51 ± 0.36	1.59 ± 0.09	1.37 ± 0.13	2.52 ± 0.06	1.05 ± 0.01	1.38 ± 0.24	1.98 ± 0.20	2.77 ± 0.11
(E)-2-Heptanal	Ald	n.d	4.35 ± 0.15	11.41 ± 0.10	10.63 ± 2.09	3.13 ± 0.07	3.52 ± 0.27	n.d	14.40 ± 0.06	17.65 ± 0.92	2.72 ± 0.20	1.01 ± 0.05	n.d	1.87 ± 0.25
Trans-2-nonenal	Ald	n.d	0.70 ± 0.13	0.75 ± 0.06	0.91 ± 0.18	n.d	n.d	n.d	n.d	0.58 ± 0.13	0.79 ± 0.08	n.d	n.d	0.82 ± 0.03
Benzaldehyde	Ald	6.14 ± 0.09	2.27 ± 0.29	2.39 ± 0.31	1.92 ± 0.48	2.93 ± 0.31	2.08 ± 0.70	1.67 ± 0.08	1.88 ± 0.16	1.63 ± 0.04	2.86 ± 0.22	2.82 ± 0.78	2.28 ± 0.16	3.87 ± 0.53
Phenylacetaldehyde	Ald	0.55 ± 0.04	n.d	n.d	n.d	n.d	n.d	n.d	0.77 ± 0.01	0.94 ± 0.13	1.29 ± 0.04	1.99 ± 0.66	1.57 ± 0.06	1.86 ± 0.18
Decanal	Ald	n.d	1.50 ± 0.20	2.09 ± 0.31	2.18 ± 0.65	1.80 ± 0.31	n.d	1.22 ± 0.04	1.66 ± 0.13	1.51 ± 0.01	1.75 ± 0.01	1.14 ± 0.38	0.65 ± 0.16	2.22 ± 0.22
Furfural	Ald	1.46 ± 0.01	2.37 ± 0.23	1.06 ± 0.30	n.d	1.52 ± 0.57	1.70 ± 0.54	n.d	0.60 ± 0.32	n.d	1.40 ± 0.27	2.93 ± 0.02	2.11 ± 0.08	3.01 ± 0.02
Heptanal	Ald	5.68 ± 0.11	4.98 ± 0.81	4.28 ± 0.24	n.d	2.89 ± 1.90	5.70 ± 0.37	n.d	2.62 ± 0.27	n.d	n.d	n.d	n.d	4.38 ± 1.93
Hexanal	Ald	38.6 ± 2.38	19.6 ± 2.17	6.15 ± 0.98	12.3± 2.24	11.5 ± 0.28	15.2 ± 2.44	13.0 ± 2.58	12.97 ± 1.35	7.23 ± 0.58	23.5 ± 0.02	29.16 ± 0.38	30.9 ± 3.53	20.8 ± 1.07
1,4,7,10,13,16-hexaoxacyclooctadecane	Et	3.84 ± 1.40	6.62 ± 0.33	5.53 ± 0.27	5.22 ± 0.03	6.57 ± 0.78	7.01 ± 0.24	5.61 ± 1.11	2.89 ± 0.23	6.29 ± 1.67	4.01 ± 0.87	1.76 ± 0.66	4.59 ± 0.74	7.48 ± 2.47
15-crown-5	Et	4.05 ± 1.96	n.d	n.d	n.d	n.d	n.d	n.d	1.59 ± 0.46	1.51 ± 0.61	3.35 ± 1.61	3.04 ± 0.80	1.53 ± 0.04	1.62 ± 0.52
18,18’-Bi-1,4,7,10,13,16-hexaoxacyclononadecane	Et	1.64 ± 0.15	5.53 ± 0.12	n.d	n.d	2.02 ± 0.07	n.d	n.d	n.d	1.38 ± 0.25	n.d	2.43 ± 1.45	n.d	2.38 ± 0.21
3,6,9,12-Tetraoxatetradecan-1-ol	Et	2.08 ± 0.83	n.d	2.89 ± 0.15	n.d	n.d	n.d	n.d	0.55 ± 0.20	2.83 ± 0.22	n.d	1.14 ± 0.03	n.d	n.d
Methoxybenzoxime	Et	2.49 ± 0.05	2.57 ± 0.58	1.52 ± 0.18	3.39 ± 1.13	3.54 ± 0.73	3.94 ± 0.18	3.54 ± 1.17	2.44 ± 0.11	2.33 ± 0.61	2.71 ± 0.38	3.49 ± 1.70	4.11 ± 0.01	2.79 ± 1.22
Tetraethylene glycol diethyl ether	Et	n.d	1.53 ± 0.05	1.35 ± 0.03	n.d	n.d	n.d	n.d	n.d	2.73 ± 0.53	1.57 ± 0.02	n.d	n.d	3.24 ± 0.48
Conjugated (10E, 12Z)-linoleic acid	Fc	n.d	n.d	0.76 ± 0.12	1.61 ± 0.08	n.d	1.65 ± 0.55	3.19 ± 0.54	n.d	2.07 ± 0.05	1.39 ± 0.03	n.d	n.d	1.57 ± 0.05
(2S,2’S)-2,2’-Bis[1,4,7,10,13-pentaoxacyclopentadecane]	E	1.73 ± 0.52	2.20 ± 0.21	n.d	3.32 ± 0.45	2.38 ± 0.88	n.d	n.d	n.d	n.d	n.d	4.17 ± 0.15	2.35 ± 0.22	n.d
3-ethyl-2-methyl-1,3-hexadiene	Hc	1.50 ± 0.34	0.79 ± 0.16	1.04 ± 0.01	n.d	n.d	n.d	1.44 ± 0.31	1.03 ± 0.33	1.02 ± 0.03	1.22 ± 0.51	n.d	n.d	n.d
6-Methyl-5-hepten-2-one	K	0.87 ± 0.21	1.66 ± 0.49	2.37 ± 0.37	2.24 ± 0.39	2.16 ± 0.02	2.13 ± 0.15	1.72 ± 0.03	2.14 ± 0.04	2.23 ± 0.18	1.75 ± 0.01	1.41 ± 0.23	1.50 ± 0.16	1.23 ± 0.46
2-pentyl-furan	Ar	2.85 ±0.43	n.d	n.d	n.d	0.98 ± 0.01	2.47 ± 0.36	n.d	n.d	n.d	4.38 ± 0.39	4.85 ± 2.01	5.11 ± 0.41	1.81 ± 0.31
(2S,13S)-12,13-Dihydroxy-1,4,7,10-tetraoxacyclotetradecane		0.58 ± 0.05	n.d	2.00 ± 0.15	3.58 ± 0.02	n.d	n.d	n.d	n.d	n.d	1.70 ± 0.37	5.65 ± 2.33	n.d	n.d
2- [2- [2- [2- [2- [2- [2-(2-(2-Hydroxyethoxy)ethoxy]ethoxy]ethoxy]ethoxy]ethoxy]ethoxy Base] ethanol		2.77 ± 0.28	7.44 ± 0.22	5.96 ± 0.53	3.47 ± 0.15	7.45 ± 2.37	3.72 ± 0.22	4.07 ± 1.24	2.95 ± 0.11	2.37 ± 0.87	n.d	2.59 ± 1.22	1.89 ± 0.07	n.d
2- [2- [2- [2- [2- [2- [2- [2- [2- [2-(2-Methoxyethoxy)ethoxy]ethoxy]ethoxy]ethoxy Ethoxy]ethoxy]ethoxy]ethoxy]ethoxy]ethanol		n.d	n.d	6.00 ± 1.33	n.d	2.19 ± 0.15	6.10 ± 1.25	1.06 ± 0.76	0.91 ± 0.22	1.85 ± 0.77	n.d	n.d	n.d	n.d
2- [2- [2- [2- [2- [2- [2- [2- [2- [2- [2- [2- [2- [2-(Trimethylsilyloxy)ethoxy Ethoxy]ethoxy]ethoxy]ethoxy		0.91± 0.02	4.66 ± 1.88	n.d	n.d	1.27 ± 0.11	5.51 ± 0.03	3.75 ± 1.27	4.26 ± 0.75	1.41 ± 0.47	2.02 ± 0.33	n.d	1.99 ± 0.05	n.d

Results are presented as mean ± standard deviations (*n* = 3). DTN–dried tea noodles; Cc—Chemical classes of volatile compounds; A—Alcohols; Ald—Aldehydes; Hc—Hydrocarbons; Et—Ethers; K—Ketones; Ar—Heteroaromatic; n.d.—not detected, M—Matcha tea powder, G—Green tea powder, B—Black tea powder; enriched (0.5–2%). CTRL: noodles without tea powder; M-0.5%, M-1.0%, M-1.5%, and M-2.0%: noodles with additional 0.5%, 1.0%, 1.5%, and 2.0% matcha tea powder, respectively; G-0.5%, G-1.0%, G-1.5%, and G-2.0%: noodles with additional 0.5%, 1.0%, 1.5%, and 2.0% green tea powder, respectively; B-0.5%, B-1.0%, B-1.5%, and B-2.0%: noodles with additional 0.5%, 1.0%, 1.5%, and 2.0% black tea powder, respectively; All addition levels were on a flour basis.

## Data Availability

Data is contained within the article.
